# Sex performance differences in vertical and horizontal jumping

**DOI:** 10.1098/rsos.241920

**Published:** 2025-09-24

**Authors:** Emily L. Haag, Peter G. Weyand

**Affiliations:** ^1^ Locomotor Performance Laboratory, Department of Applied Health Sciences, Texas Christian University, Fort Worth, TX, USA; ^2^ Locomotor Performance Laboratory, Department of Kinesiology, Texas Christian University, Fort Worth, TX, USA

**Keywords:** body composition, gravity, kinetics, force application, mass-specific force

## Abstract

Performance differences between males and females are substantially greater when humans jump for maximal height versus distance. We postulated that the lower muscle mass/body mass fractions of females would cause sex differences in performance to increase as jump take-off angle and the force required to elevate body mass against gravity (force/body weight) increased. We tested this idea using triple jump (TJ), long jump (LJ) and high jump (HJ) data from World Athletics best-performers lists (*n* = 40 per sex) and countermovement jump (CMJ) data acquired from collegiate athletes (*n* = 19 per sex) jumping from force platforms. Across the four jumps, the more vertically oriented the take-off angle (θ_TO_), the greater the sex difference observed [range: 17.4–42.1% from TJ to CMJ; regression equation: %Diff = 26.9 (sinθ_TO_)^2^ + 14.2, *R*^2^ = 0.98]. For the strictly vertical CMJ, between-sex differences in jump height (∆ = 42.1%) were eight times larger than the differences in ground-phase force application (∆force/body mass = 5.1%). We conclude that (i) small differences in mass-specific ground force application result in much larger differences in performance for more vertically oriented and gravity-opposed jumps, and (ii) lower muscle mass/body mass fractions require females to use more of their available force to offset gravity, thereby leaving them with less remaining force to elevate body mass.

## 1. Introduction

The relationship between whole-body biological factors and jumping performance has attracted attention dating to the classical work of several pioneering investigators [1–3]. These early investigators focused primarily on the body mass–jump height relationship, with each independently concluding that maximal jump heights should be largely independent of body mass. However, relatively little attention has been devoted to the influence of body composition. Because jumping performance is directly influenced by the forces jumpers can apply to the ground, the proportion of the body’s mass comprised of skeletal muscle probably influences the mass-specific ground reaction forces that determine bodily motion and performance. At present, a quantitative understanding of this factor is not available.

Humans are an opportune species for investigating potential linkages between body composition and jumping performance for a number of reasons. First, in contrast to most species for whom quality jumping performance data are available, humans are highly dimorphic by sex in body composition, particularly in muscle/body mass fractions. Second, the relevant sex differences in body composition have been extensively quantified and are, therefore, well established; proficient male jumpers typically have muscle/body mass fractions that are 20% greater than females [4,5]. Third, extensive, high-quality human data are available for a variety of jump types.

One noteworthy feature of human jumping performance data is that the influence of muscle/body mass fractions appears to differ by jump type. The smallest sex differences for human jumpers are generally reported for horizontal jumps, such as the long and triple jumps, that have relatively shallow jump trajectories. In these events, male athletes typically jump 15–18% [6,7] farther than equivalently athletic females. The gaps reported for horizontal jumps initiated from a standstill (i.e. the standing broad jump) that have more vertical jump trajectories than the run-in horizontal jumps are substantially larger. Studies generally report distances that are 20–35% greater for adult male versus female populations [8,9]. In strictly vertical jumps initiated from a standing position, like the squat and countermovement jump, between-sex performance gaps are larger still. Adult males typically attain maximal heights that are 25–50% higher than adult females [8,10–16]. Additionally, the sex performance differences for jumps, regardless of type, are substantially greater than those for events without vertical requirements, such as track running events [6,7].

Here, we postulated that the force required to overcome gravity might explain why sex performance differences are larger for more vertically oriented jumps. The relatively small muscle/body mass fractions of female jumpers would theoretically require females to use more of their available force to offset gravity, leaving them with less force remaining to elevate the body’s mass. Accordingly, here we tested the hypothesis that the sex difference in jumping performance would be greater for more vertically oriented jumps. We did so by examining sex performance differences in four jump types chosen for their differing requirements for horizontal vs. vertical motion: triple jump (TJ), long jump (LJ), high jump (HJ) and countermovement jump (CMJ).

## 2. Methods

The first portion of this section provides the theoretical framework we used to make *a priori* estimates that relate sex-specific ground force/mass capabilities to vertical jumping mechanics. The second portion presents our data acquisition and hypothesis testing procedures for analysing the four jump types expected to differ in their requirement for vertical motion. For this purpose, we used available high-quality performance data from World Athletics for three Olympic jumping events (long, triple and high jumps). For the exclusively vertical countermovement jump, we acquired literature and original laboratory data from equivalently athletic male and female athletes.

### 2.1. Part I: theoretical framework

#### 2.1.1. Mechanics of vertical jumping

The effects of gravity on the ground reaction force available to accelerate the jumper initially, and the body’s aerial motion, subsequently, are direct determinants of jump height. The example that follows provides a theoretical explanation for how small differences in force-generating capacity and ground force application (force/mass) could translate into much larger differences in performance (here, jump height). We do so for the CMJ using equation (2.1), where *F*_z-total_ is the total vertical ground reaction force measured, *F*_Wb_ is the force of gravity on the body’s mass that must be overcome and *F*_z-net_ is the force available to accelerate the body’s mass vertically.


(2.1) $$F_{z-net}=F_{z-total}–F_{Wb}$$

Hypothetically, using equation (2.1) for male and female jumpers who apply respective total vertical forces (*F*_z-total_) of 2.0 and 1.8 × body weight, during the ground phase (i.e. countermovement plus push-off periods), the 10% difference in the *total* mass-specific ground force applied will correspond to a 20% difference in the *net* force available to accelerate the body vertically (male: *F*_z-net_ = 2.0 – 1.0 = 1.0; female: *F*_z-net_ = 1.8 – 1.0 = 0.8; ~ 20% difference).

After leaving the ground to become airborne, gravitational effects will result in any sex-specific differences in the ground phase kinetics becoming exponentially larger differences in jump height. This follows simply from the different time–displacement relationships for horizontal versus vertical projectile motion, provided by equation (2.2) for horizontal and equation (2.3) for vertical displacement below. Horizontal displacement (*s*_y_) at any given time (*t*) after take-off is a linear function of the take-off velocity (∝*V*_y-TO_, equation (2.2)). Whereas the maximal vertical displacement (*s*_z_, or height attained after take-off) is a function of the take-off velocity squared (∝ $V_{z-TO}^{2}$, equation (2.3)) and gravity (*g*).


(2.2) $$s_{y}=V_{y-TO}\;∙\;t$$

(2.3) $$s_{z}=\frac{V_{z-TO}^{2}}{\left(2g\right)}$$

#### 2.1.2. Sex-specific force/mass estimates

The muscular forces available to a jumper are a function of the physiological cross-sectional area of the limb extensors (force capability). Genetic factors endow males with relatively less body fat and relatively more skeletal muscle than females, with differences that are slightly less pronounced for the lower versus upper body [4,5,17,18]. To approximate the effect of sex differences in body composition on mass-specific force capabilities, we estimated *a priori* sex-specific force/mass capability from an overall ratio of lower-limb extensor muscle physiological cross-sectional area to body mass for both male and female athletes. This estimate is based on the well-established differences in the tissue make-up of male and female bodies, lower body limb extensor muscle masses and representative fibre lengths [18,19] applied to the average masses of our male and female CMJ samples (87.0 ± 9.8 and 70.7 ± 7.7 kg, respectively; table 1, estimated area/*M*_b_).

**Table 1.** Measured and estimated anatomical characteristics of CMJ subjects

	male (*n* = 19)	female (*n* = 19)
height (m)	1.85 ± 0.08	1.73 ± 0.06
mass (kg)	87.0 ± 9.84	70.7 ± 7.70
estimated % fat	10.0	20.0
estimated % FFM *M*_sm_	60.0	54.4
estimated LL *M*_sm_/*M*_b_	0.297	0.253
estimated area/*M*_b_ (cm^2^ kg^−1^)	0.038	0.034

Values are mean ± s.d., estimated % FFM *M*_sm_: fat free mass made up of skeletal muscle, estimated LL *M*_sm_/*M*_b_: lower limb skeletal muscle mass/*M*_b_ ratio, estimated area/*M*_b_: overall lower-limb physiological cross-sectional area/body mass ratio.

We incorporated body fat percentages of 10% and 20% for males and females, respectively, and estimates of 60.0% and 54.4% of respective fat-free body mass made up of skeletal muscle [4,5] to estimate total skeletal muscle masses. Next, magnetic resonance imaging data of the body’s total skeletal muscle mass in the lower bodies of males and females [17] were used to quantify total lower-limb skeletal muscle mass. Ratios of lower limb skeletal muscle mass to total body mass ratios were then determined (table 1, estimated LL *M*_sm_/*M*_b_). Lower limb-skeletal muscle mass estimates were divided by the average lower limb muscle fascicle lengths from cadaveric data (0.0781 cm; [19]) to obtain an estimated physiological cross-sectional area of the lower body skeletal muscle. These steps led to the overall physiological cross-sectional area of lower-limb skeletal muscle to body mass ratio estimates appearing in table 1 (estimated LL area/*M*_b_). Our analysis indicated males should have a lower-limb muscle area/body mass ratio and thus force/mass jumping capabilities approximately 9.5% greater than females.

Our theoretical framework led us to the following *a priori* expectations: (i) a baseline between-sex difference of approximately 10% in the ground force applied per unit body mass that would generalize across all four jump types, including our original data on equivalently athletic samples of male and female CMJ subjects, and (ii) between-sex jump performance differences that are greater for jumps with more vertically oriented take-off angles.

### 2.2. Part II: experimental design and hypothesis testing

#### 2.2.1. Literature data

**Figure 1.**
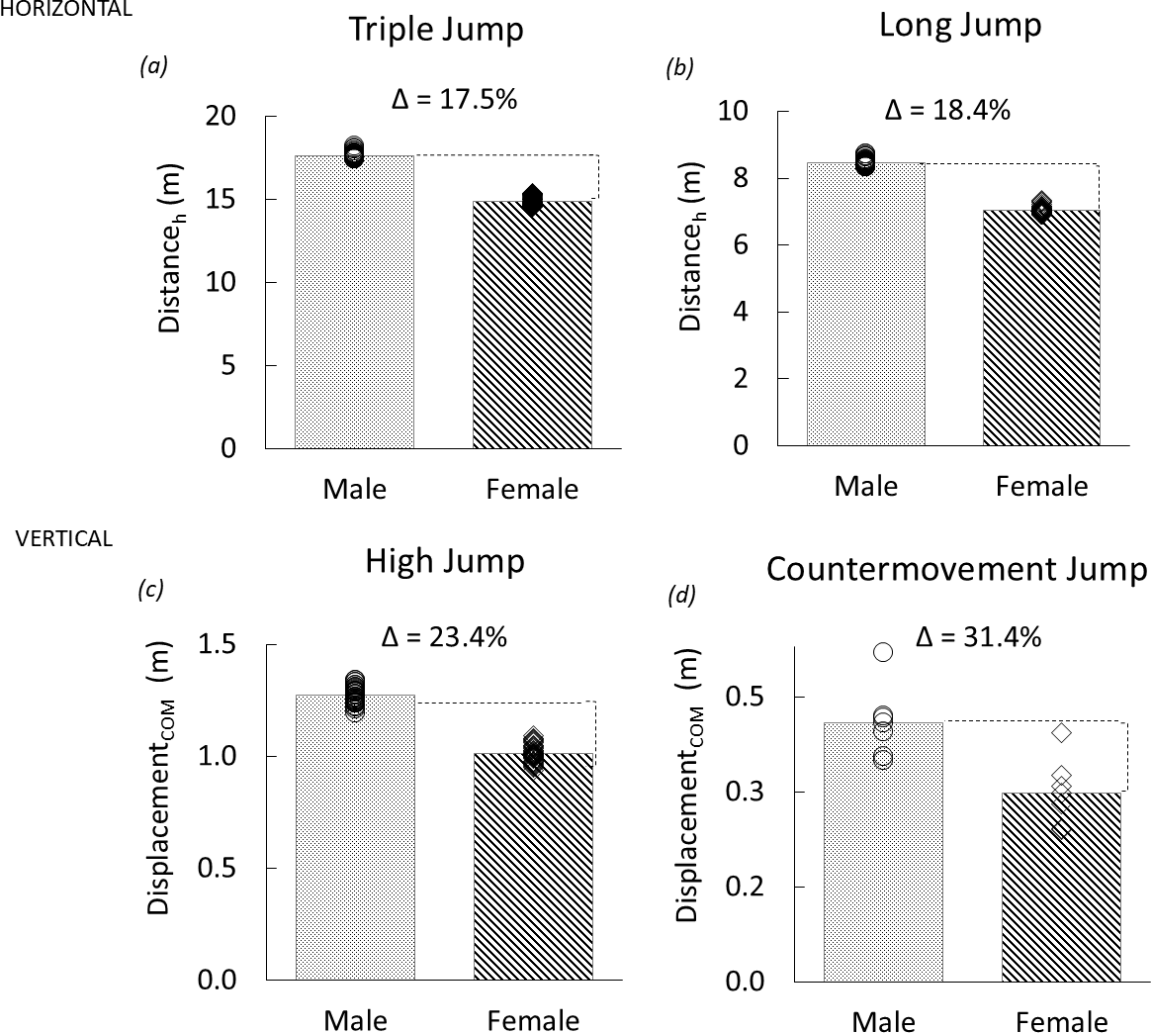
Male and female literature performance data for horizontal and vertical jumps: TJ (*a*, *n* = 40 per sex, [20]) and LJ (*b*, *n* = 40 per sex, [20]) distances and estimated centre of mass (COM) displacement for the HJ (*c*, *n* = 40 per sex, [20]), and CMJ for Norwegian National Team members (*d*, *n* = 6 group means per sex, [15]). The corresponding bars are means of individual performance data noted by circles for males and diamonds for individual females, excluding the CMJ data, where each symbol is an athletic group mean. [Due to size insufficiency, s.d. are not displayed. Values are as follows: (*a*) male s.d. ± 0.18, female s.d. ± 0.26 m, (*b*) male s.d. ± 0.10, female s.e. ± 0.11 m, (*c*) male s.d. ± 0.04, female s.e. ± 0.04 m, (*d*) s.d. ± 0.06, female, s.d. ± 0.06 m.]

Performances in the three Olympic jumps (TJ, LJ and HJ) were analysed using publicly available data from the top male and female performers (*n* = 40 males, *n* = 40 females, per event) over a 15-year period (2003−2018) from World Athletics’ top lists [20] to analyse sex performance differences. Take-off angles were not available for individual performers and were therefore quantified as an average of the angles reported from the sources presenting these data for groups of male and female jumpers in World Championship competitions for the respective jumps: TJ, [21–25], LJ [21,24,25] and HJ [21,24,25]. Final TJ values represent the average of the three within-event jumps. Centre of mass velocities at take-off for each event were also acquired and reported as averages of the available data (TJ: [24,25]; LJ: [24,25]; HJ: [24]; table 2). Velocity and take-off angle data acquired from the literature also appear in table 2. The mean take-off angles for male and female athletes for the three Olympic jumps agreed with one another closely (within 0.3°, 1.4° and 4.0° for the TJ, LJ and HJ, respectively; [21–25]). As such, the same take-off angles were used for males and females for each jump in the analysis; these angles appear in table 2. Finally, for acquiring a literature baseline sex performance difference for the non-Olympic CMJ, data were acquired from Norwegian National Team athlete groups reported by Haugen *et al*. [15] and are presented in figure 1.

**Table 2.** COM take-off angles and velocities.

take-off angle				take-off velocity				
event	take-off angle (degrees)	sample size (M, F)	source	male ± s.d. (m s^−1^)	female ± s.d. (m s^−1^)	sex difference (%)	sample size (M, F)	source
triple jump	16.4	44, 37	[21–25]	8.12 ± 0.29	7.29 ± 0.29	10.8	27, 29	[24,25]
long jump	22.3	30, 28	[21,24,25]	9.44 ± 0.39	8.55 ± 0.42	9.9	27, 25	[24,25]
high jump	40.9	22, 13	[21,24,25]	6.27 ± 0.33	5.71 ± 0.31	9.4	10, 12	[24]
countermovement jump	90	19, 19	present study	3.28 ± 0.37	2.65 ± 0.30	21.3	19, 19	present study

COM velocity values are resultants for all four jumps. Take-off angles were the same for males and females (see §2.2.1).

*Olympic jump performance metrics*. For both triple and long jumps, between-sex performance differences were quantified using the measured jump distance reported from international competitions. For the high jump, the standing height of the centre of mass, which is lower in females than in males, was subtracted from the bar clearance height to better represent the jump centre of mass elevation. The standing height of each athlete’s centre of mass (COM) was estimated from equations formulated from male and female cadavers (male COM = stature × 0.568; female COM = stature × 0.554 [26]). Heights and weights for individual athletes in the performance data samples were gathered from publicly available sources [27,28].

#### 2.2.2. Experimental countermovement jump data acquisition

Original countermovement jump data were acquired from male (*n* = 19; height: 1.85 ± 0.08 m, mass: 87.0 ± 9.8 kg) and female (*n* = 19; height: 1.73 ± 0.08 m, mass = 70.7 ± 7.7 kg) volunteers who had competitive collegiate athletic experience in one or more of the following sports: soccer, tennis, track and field, baseball, softball, volleyball, swimming, diving, lacrosse and American football. All volunteers were competing and/or physically active at the time of testing and provided written, informed consent in accordance with protocol 2013-H20 approved by the Institutional Review Board of Southern Methodist University.

Force platform data were acquired from a Bertec force plate system, consisting of three FP9090-15-4000 (0.9 × 0.9 m) plates, collected at 1000 Hz. Signals were processed through Bertec AM6800 amplifiers and a National Instruments PCIe-6323 data acquisition board (32 analogue inputs, 250 kS s^−1^, 16-bit resolution). Custom LabVIEW 2011 software was used to implement the data acquisition protocol.

Participants were outfitted with standard laboratory compression shorts and T-shirts, as well as standardized shoes (Brooks; Cascadia 8). After a short, standardized warm-up, a minimum of three practice jumps, and a 10 s standing trial, participants performed three maximal effort vertical jumps from the force platforms. Subjects were instructed to jump as high as possible in the three trials with instruction to take the time between trials needed for full recovery.

CMJ heights were determined using vertical velocity at take-off (*V*_z-TO_) calculated from the vertical impulse of the jump using the impulse–momentum relationship (equation (2.4); where: *F*_total_ = total vertical ground reaction force (N), *F*_Wb_ = force of body weight (N), ∆*t* = time of force application (s, pre-countermovement through take-off), *M*_b_ = body mass (kg), *V*_z-TO_ = vertical velocity at take-off (m s^−1^)).


(2.4) $$V_{z-TO}\;=\frac{\left(F_{total}\;-F_{Wb}\right)∙\Delta t}{M_{b}}.$$

*V*_z-TO_ was then applied to calculate the displacement of the jumper’s COM (*s*_z_) or jump height (m) using the equation for vertical projectile motion provided earlier (equation (2.3)).

Jump time was determined using a 40 N deviation from the subject’s standing baseline ground reaction force plus 0.25 s to ensure valid, consistent impulse measurement across subjects. Take-off was defined as the first instant at which the vertical force was less than 40 N. Each participant’s highest jump of the three was used in subsequent analysis.

Throughout the manuscript, we describe the action force the jumper applies to the ground as the applied force (and/or the act of ground force application), and the force measured from the in-ground force platform as the ground reaction force. In the context of jumping, the terms ‘force’ and ‘jumping force’ are generally used to refer to both the action and reaction forces involved in the jump.

### 2.3. Data and statistical analysis

Between-sex differences in jumping performances for TJ, LJ, HJ and CMJ were quantified on a percentage basis. For each of these variables and throughout the manuscript percent differences were calculated as


(2.5) $$sex\;difference=\left(\frac{male_{avg}-female_{avg}}{\left(\left(male_{avg}+female_{avg}\right)/2\right)}\right)\times \;100.$$

A 2 × 4 ANOVA was used to test for an interaction of sex and jump type on normalized performance scores for male and female performances including the TJ, LJ, HJ and the literature CMJ data. Unpaired *t*-tests were used to compare male and female performances in all four jump types. An unpaired *t*‐test was also used to compare male and female CMJ performance heights. A regression model assuming a squared relationship was used to fit the relationship between sex performance difference (%) and the sine of the take-off angle (*θ*_TO_) across the four jump types. An overall *α* level was set at 0.05.

## 3. Results

### 3.1. Performance data from literature sources

Mean performances and sex performance differences from the literature for male and female athletes for the four jump types appear in figure 1. Individual performances for the Olympic jumps are plotted as circles (male) and diamonds (females). The CMJ data points (circles and diamonds) are athlete group averages from Haugen *et al.* [15] for male and female Norwegian National Team athletes. Across the four jump types, sex performance differences were systematically greater for the jumps with more vertical take-off angles. Percent difference values for the literature data spanned a nearly twofold range from 17.5% for the TJ to 31.4% for the CMJ data of Haugen *et al.* (figure 1*a*–*d*). A significant interaction was observed between sex and jump type for the performance differences for the four events, ranging from least (TJ) to most (CMJ) vertical (*F*_(1,3)_ = 15.571, *p* < 0.001). Mean sex performance differences for each event were also significant for all pairwise comparisons (*p* < 0.001). Mean take-off angles for the four jumps ranged from 16.4° to 90°, with the following rank ordering from least to most vertical: TJ, LJ, HJ and CMJ (table 2).

### 3.2. Laboratory countermovement jump data

#### 3.2.1. Individual data

**Figure 2.**
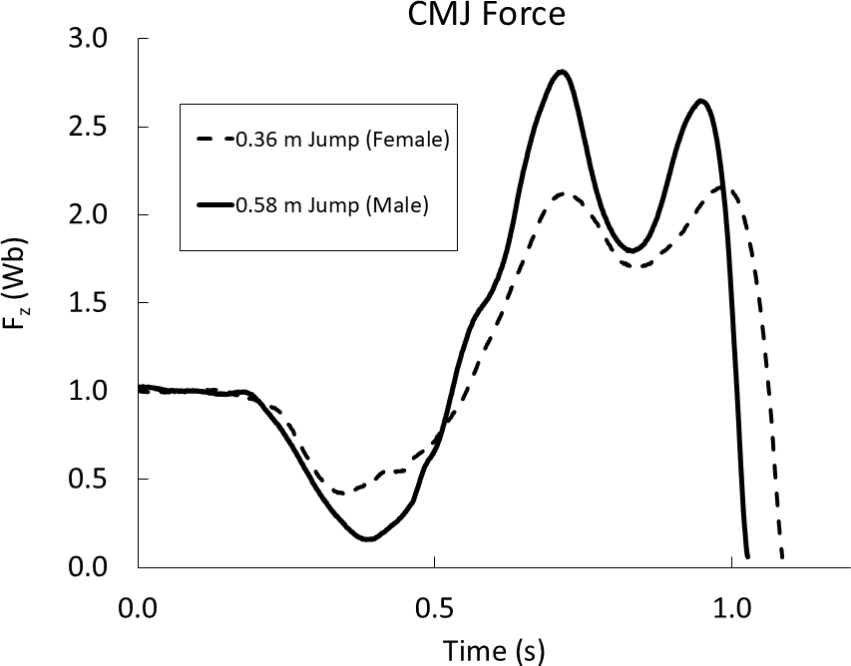
Vertical force (*F*_z_) in body weights (*W*_b_) versus time (s) during the ground phase of a maximal effort CMJ from standing to take-off for one female (dashed line) and one male (solid line) participant. Corresponding jump kinetic metrics are provided in table 3.

Representative force–time data from one male and one female participant who performed the CMJ in the laboratory, with force standardized using body weight units (*W*_b_), appear in figure 2. The male participant had greater mass-specific vertical ground reaction forces throughout the ground phase while the time of force application was similar for the two jumpers. The total force, net force, impulse, take-off velocity and jump height values corresponding to the respective waveforms in figure 2 appear in table 3. The percent difference between the male and female subjects for *total* mass-specific force of 6.1% was substantially smaller than the corresponding differences in *net* mass-specific force, impulse and take-off velocity which ranged from 23.0% to 28.4%. The jump height difference between these two subjects was appreciably greater at 46.8%.

**Table 3.** CMJ kinetics for one male and one female subject.

	male	female	difference (%)
*F*_z-total_ (*W*_b_)	1.33	1.25	6.4
*F*_z-net_ (*W*_b_)	0.33	0.25	28.4
impulse (*W*_b_ × s)	0.34	0.27	23.0
*V*_z-TO_ (m s^–1^)	3.37	2.68	23.0
jump height (m)	0.58	0.36	46.8

Values correspond to the force-time waveform data appearing in figure 2.

#### 3.2.2. Group mean data

**Figure 3.**
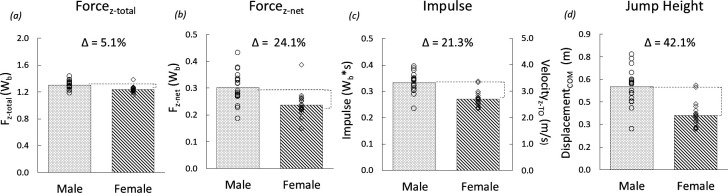
Mean total (*a*), and net (*b*), vertical ground reaction force in body weights (*W*_b_), vertical impulse in body weight seconds (*W*_b_·s) and resulting vertical take-off velocity (*c*, m s^–1^), and jump height (*d*, m) for the CMJ by collegiate athletes (*n* = 19 male [circles] *n* = 19 female [diamonds]). [Due to size insufficiency, s.d. are not displayed. Values are as follows: (*a*) male s.d. ± 0.06, female s.d. ± 0.05 *W*_b,_ (*b*) male s.d. ± 0.06, female s.d. ± 0.05 *W*_b,_ (*c*) male s.d. ± 0.04, female s.d. ± 0.03 *W*_b_·s, male s.d. ± 0.37, female s.d. ± 0.30 m s^–1^, (*d*) male s.d. ± 0.12, female s.d. ± 0.08 m.]

Mean data from countermovement jumps executed for the male and female athletes tested on the laboratory force platform appear in figure 3. The mean between-sex difference in *F*_z-total_ was relatively small (5.1%, figure 3*a*), while the difference in *F*_z-net_ was larger at 24.1% (figure 3*b*). Because the time of force application (male: 1.13 ± 0.12, female: 1.17 ± 0.22 s) was nearly identical, differences in the impulse and *V*_z-TO_ (21.3%, figure 3*c*) closely agreed with the differences in *F*_net_ (24.1%, figure 3*b*). The sex difference in *V*_z-TO_ was 21.3%, ultimately resulting in a 42.1% sex difference in jump height (figure 3*d*).

### 3.3. Sex performance differences versus take-off angle

**Figure 4.**
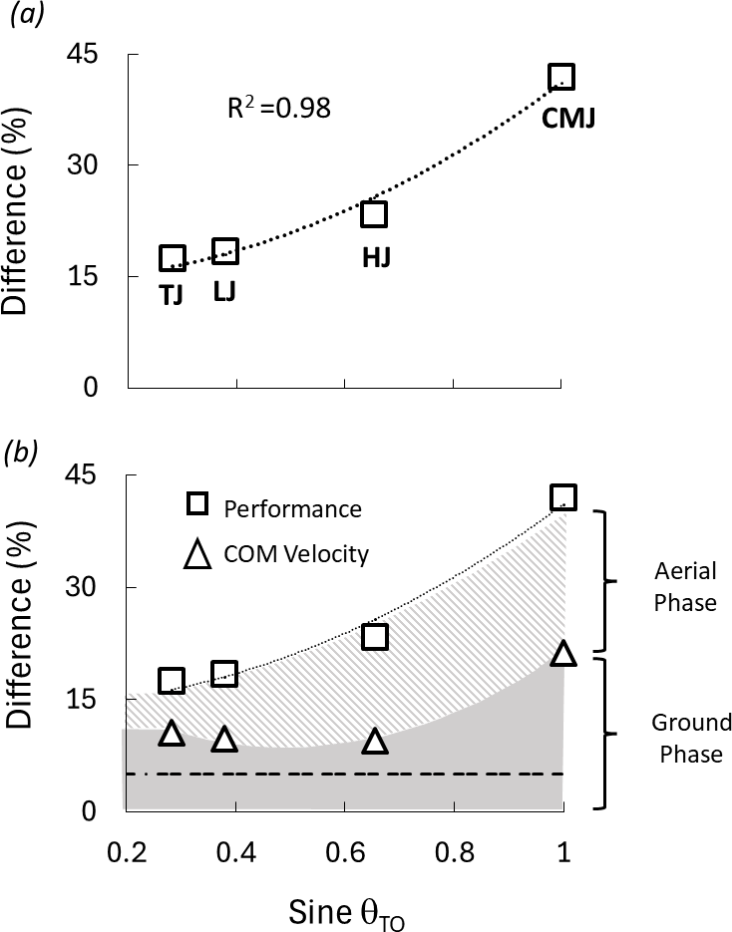
Mean sex differences (%) in performance (squares, *a* and *b*) and centre of mass (COM) velocities at take-off (triangles, *b*) for TJ, LJ, HJ and CMJ as a function of the sine of the take-off angle (sine θ_TO_). The dashed line represents the CMJ sex difference of 5.1% measured for the mass-specific ground reaction force. The estimated portion of the sex performance difference corresponding to COM velocity differences at take-off is shaded grey; the estimated portion attributed to the effect of gravity on airborne COM trajectories is the diagonally hatched area (*b*). [Corresponding COM velocity means (m s^−1^) and take-off angles (°) appear as numeric values in table 2]. Note: COM velocity data were resultants for all four jumps. The regression equation appearing in panel *a* is: Y=26.9(x)^2^ +14.2.

A regression model of the sine of the take-off angle squared accounted for nearly all of the sex performance differences present across the four jump types (figure 4*a*, sex difference (%) = 26.5(sinθ_TO_)^2^ + 14.2, *R*^2^ = 0.98 (*F*_(1,3)_ = 149.57, *p* < 0.05).

Between-sex percent differences in both take-off velocities and jump performance in relation to the sine of the take-off angle appear in figure 4*b*. For the four jump types, the percentage difference in performance was greater in every case than the percentage difference in take-off velocity. The difference between the two increased as a function of the sine of the jump take-off angle, being relatively small for the TJ (7% = 17 versus 10), slightly larger for the LJ (10% = 18 versus 8), moderately large for the HJ (14% = 23 versus 9) and largest for the CMJ (21% = 42 versus 21). The net vertical impulse during the ground phase accounted for just over half of the total difference in height (21.3% per figure 3*c* versus the total difference of 42.1%) while the gravitational acceleration of the body in the air accounted for the remainder (20.8% of the 42.1% performance difference).

## 4. Discussion

Our hypothesis that sex differences in body composition would result in larger sex performance differences for more vertically oriented jumps was fully supported by our analysis. Across the four jump types examined, the take-off angles used by jumpers of both sexes were similar, ranging from minimums of less than 20° for the TJ to maximums of 90° for the fully vertical CMJ. The corresponding mean sex performance differences also spanned a broad range, from a minimum of 17.5% for the largely horizontal TJ to a maximum of 42.1% for the fully vertical CMJ. Our analysis of the relationship between the two indicated that nearly all of the performance variation by sex was explained by the take-off angle of the jump (sine *θ*_TO_, figure 4; *R*^2^ = 0.98).

The broad range of jumping sex performance differences we observed is most easily understood by initially considering our CMJ ground reaction force and performance data (figure 3, equations (2.1), (2.3) and (2.4)). Per both our theoretical framework and ground reaction force data, females were only marginally less forceful than males in the *total* ground forces they applied for this jump (Δ = 5.1%, *F*_z-total_, ∆force/body mass, equations (2.1), figures 2 and 3). However, the small difference in mass-specific ground force application translated into jump height differences that were more than eight times larger (∆ = 42.1%). From a purely mechanical perspective, as we detail below, this outcome is explained by the opposing effect of gravity on motion and displacement that is fully vertical (equations (2.1) and (2.3), figure 4). Because the horizontal component of the body’s motion is not opposed by gravity (equation (2.2)), performance for those jumps with more horizontal take-off angles and trajectories are less resisted, and therefore less affected. Thus, the smaller sex performance differences we observed at more horizontal take-off angles would therefore be expected for any given difference in the total mass-specific ground forces applied.

### 4.1. From small differences in mass-specific force to large differences in performance

Established sex differences in body composition and muscle/body mass fractions led us to expect that our male athletes would apply greater jumping ground forces than our female athletes (table 1). For the CMJ, the mean ground reaction forces we measured for the males were indeed greater, but by only 5.1%. How did this small sex difference in ground force application bring about a 42.1% difference in CMJ height? This result is explained by the magnitude of the gravitational force (figures 2 and 3; table 3) opposing the body’s vertical motion, initially on the ground and subsequently in the air.

The mean ground reaction forces measured over the entire force application period from standing through the countermovement phase, and up to the instant of take-off, were relatively low. For both sexes, these forces were only slightly above the body’s weight (less than or equal to 1.30 · *F*_Wb_, figure 3; males: 1.30 ± 0.06 versus females: 1.23 ± 0.07 *W*_b_). Consequently, when we subtracted body weight (i.e. *F*_z_ = 1.0) from the *total* ground reaction force applied to the ground, in order to quantify the remaining, or *net* force (*F*_z-net_) that elevated the body vertically for the jump, the resulting between-sex difference of 24.1% (males: 0.30 ± 0.06 versus females: 0.23 ± 0.07 *W*_b_) was nearly five times larger than the 5.1% difference in the *total* force applied (*F*_z-total_). Thus, the requirement that athletes devote three-quarters or more of their limb extensor force to offsetting gravity when jumping vertically resulted in relatively small sex differences in the *total* mass-specific ground reaction force (*F*_z-total_), being much larger differences in the *net* forces (*F*_z-net_, figure 3*a*–*d*). Because the time of ground force application differed little between our male versus female subjects (1.13 ± 0.12 versus 1.17 ± 0.22 s, respectively), sex differences in jump impulses that determine take-off velocities (equation (2.4)) were nearly equal to the sex difference in *net* force (24.1% versus 21.3%, figure 3*b*,*c*).

The jump height ultimately attained during the aerial period is the product of the time of the ascent to the jump apex and the average velocity during that ascent. Since both variables are a direct function of the take-off velocity, the height attained is a function of the take-off velocity squared (equation (2.3)). Here, these projectile motion relationships resulted in the 21.3% sex difference in CMJ take-off velocity (figure 3*c*; 3.28 ± 0.37 versus 2.65 ± 0.63 m s^−1^, male versus female, respectively) translating into a 42.1% difference in jump height (figure 3*d*; 0.55 ± 0.12 versus 0.36 ± 0.09 m, male versus female, respectively).

Thus, in the male and female athletes we tested, the respective ground and aerial phase contributions to the total sex difference in CMJ performance (jump height; figure 4*b*) attributable to the gravitational forces opposing the body’s vertical motion were nearly equal at 51% and 49%, respectively (ground: 21.3/42.1 = 50.6%; aerial = 20.8/42.1 = 49.4%: grey shaded and hatched portions of figure 4*b* respectively).

### 4.2. Variation in sex performance differences across the four jump types

Our CMJ jump analysis illustrating how gravitational effects can transform relatively small differences in force/mass capabilities into much larger differences in vertical jump performance also explains why sex performance differences were so closely related to the sine of the take-off angle. The sine function, as implemented here, quantifies the vertical component of the body’s motion at the instant of take-off. Consequently, this variable also quantifies the extent to which the ground reaction forces, and the body’s projectile motion in the air, are influenced by the gravitational effects quantified above for the fully vertical CMJ. When considered in this context, the larger sex performance differences observed at more vertical take-off angles (figure 4), from the measured baseline difference in ground reaction forces of 5.1% to values approaching 20% for horizontal jumps, above 20% for the HJ and above 40% for the vertical-only CMJ, are mechanistically logical in terms of gravitational effects (figure 4*b*).

The respective net ground force and airborne trajectory contributions appearing in figure 4*b* were directly quantified for the CMJ for which we had original ground reaction force and impulse data needed to do so. The values for the three Olympic jumps are approximations for several reasons. For the three Olympic jumps, neither the incoming velocities prior to take-off nor ground reaction force data were available for all the individual jumpers. In addition, the take-off angle, velocity and performance data analysed were not from identical subject groups (table 2).

However, three aspects of the results appearing in figure 4*b* are noteworthy in terms of their general alignment with our theoretical framework. First, the between-sex difference in total mass-specific ground reaction force (*F*_z-total_) for the jump initiated from a standstill (CMJ: 5.1%) was actually smaller than our theoretical expectation of a sex difference of just under 10% in the *total* mass-specific force capability during a single maximal dynamic push on the ground. Second, for each of the four jumps, the requirement for vertical motion brought about sex differences in performance that were larger than the respective sex differences in take-off velocities. Third, the magnitude of the differential between the sex difference in take-off velocity and the sex difference in performance widened as the jump take-off angle increased and the jump motion became more vertical (hatched area, figure 4*b*). Per our CMJ analysis and the classical trajectories of projectile motion, these observations are attributable to gravity affecting the vertical, but not the horizontal motion of the aerial phase of these jumps.

### 4.3. Sex performance differences in other jumping populations

We intentionally limited our analysis here to highly athletic male and female populations as a design strategy for minimizing performance variation that could result from factors other than sex. These include skill level and potentially non-representative morphological variation in non-specialized populations that could skew performance data. Nonetheless, the trend we identify here for sex performance differences to be greater for more vertically versus horizontally oriented jumps is present throughout the literature on adult populations, often with greater variation within each jump type in non-specialists.

Results from other populations of high-calibre athletes whose performances have been quantified for Olympic and countermovement jumps agree well with those we report here [6–8,11,13–16]. Similarly, the sex difference in CMJ performance incorporated here for the Norwegian National Team athletes [15] of 31% was in reasonable agreement with the 42% we measured in our own athletic test population. For less-specialized populations for whom both vertical and horizontal jump performance data are available, sex performance differences are consistently larger for the vertical versus horizontal jump data reported [8,9,12]. Finally, the sex differences for vertical jumps executed from a standstill that are available across a broad spectrum of specialized and non-specialized populations are large, ranging from as low as 23% [12] to above 50% [13].

## 5. Concluding remarks

We started this investigation with the idea that having relatively less muscle would require females to use more of the force they have available for jumping to offset gravity, thereby leaving them with less remaining force to elevate the body. Our results supported this idea in full, enabling us to explain sex performance differences that spanned a nearly 2.5-fold range from the differing take-off angles and bodily elevation requirements of the jump types examined. Extending considerations of this idea to weight-bearing performances that involve almost no vertical motion provides additional support for its general validity. The sex performance differences for standard track running events, all of which involve the same predominantly horizontal bodily trajectories, are: (i) largely the same across seven different event distances [29], and (ii) substantially smaller than the minimums we report here for jumping.

We conclude that differences in mass-specific force capabilities have greater performance consequences for more vertically oriented and gravity-opposed efforts to move the body’s mass.

## Data Availability

The data used to analyse Olympic-style jumping reported are publicly available, with citations provided. Individual kinetic data for the original countermovement jumps is provided as a supplementary file [30].

## References

[1] Hill AV. 1950 The dimensions of animals and their muscular dynamics. Sci. Prog. **38**, 209–230.

[2] Borelli GA. 1680 De motu animalium; P Maquet, trans, 1989, On the movement of animals. Berlin, Springer-Verlag.

[3] Haldane JB. 1926 On being the right size. Harper’s Magazine **152**, 424–427.

[4] Prior BM, Modlesky CM, Evans EM, Sloniger MA, Saunders MJ, Lewis RD, Cureton KJ. 2001 Muscularity and the density of the fat-free mass in athletes. J. Appl. Physiol. **90**, 1523–1531. (10.1152/jappl.2001.90.4.1523) 11247955

[5] Pipes TV. 1977 Body composition characteristics of male and female track and field athletes. Res. Q. **48**, 244–247. (10.1080/10671315.1977.10762181) 266248

[6] Thibault V *et al*. 2010 Women and men in sport performance: the gender gap has not evolved since 1983. J. Sports Sci. Med. **9**, 214–223. 24149688 PMC3761733

[7] Ospina Betancurt J, Zakynthinaki MS, Martínes-Patiño MJ, Cordente Martinez C, Rodríguez Fernández C. 2018 Sex-differences in elite-performance track and field competition from 1983 to 2015. J. Sports Sci. **36**, 1262–1268. (10.1080/02640414.2017.1373197) 28862921

[8] Peterson MD, Alvar BA, Rhea MR. 2006 The contribution of maximal force production to explosive movement among young collegiate athletes. J. Strength Cond. Res. **20**, 867–873. (10.1519/00124278-200611000-00024) 17194245

[9] Davies BN, Greenwood EJ, Jones SR. 1988 Gender difference in the relationship of performance in the handgrip and standing long jump tests to lean limb volume in young adults. Eur. J. Appl. Physiol. Occup. Physiol. **58**, 315–320. (10.1007/BF00417269) 3220073

[10] Alegre LM, Lara AJ, Elvira JL, Aguado X. 2009 Muscle morphology and jump performance: gender and intermuscular variability. J. Sports Med. Phys. Fit. **49**, 320. 19861940

[11] Philpott LK, Forrester SE, van Lopik KA, Hayward S, Conway PP, West AA. 2021 Countermovement jump performance in elite male and female sprinters and high jumpers. Proc. Inst. Mech. Eng. Part P **235**, 131–138. (10.1177/1754337120971436)

[12] Harry JR, Barker LA, Paquette MR. 2019 Sex and acute weighted vest differences in force production and joint work during countermovement vertical jumping. J. Sports Sci. **37**, 1318–1326. (10.1080/02640414.2018.1557825) 30558481

[13] Ziv G, Lidor R. 2010 Vertical jump in female and male basketball players—a review of observational and experimental studies. J. Sci. Med. Sport **13**, 332–339. (10.1016/j.jsams.2009.02.009) 19443269

[14] Laffaye G, Wagner PP, Tombleson TIL. 2014 Countermovement jump height: gender and sport-specific differences in the force-time variables. J. Strength Cond. Res. **28**, 1096–1105. (10.1519/jsc.0b013e3182a1db03) 23838969

[15] Haugen TA, Breitschädel F, Wiig H, Seiler S. 2021 Countermovement jump height in national-team athletes of various sports: a framework for practitioners and scientists. Int. J. Sports Physiol. Perform. **16**, 184–189. (10.1123/ijspp.2019-0964) 33217727

[16] Castagna C, Castellini E. 2013 Vertical jump performance in Italian male and female national team soccer players. J. Strength Cond. Res. **27**, 1156–1161. (10.1519/jsc.0b013e3182610999) 22692110

[17] Janssen I, Heymsfield SB, Wang Z, Ross R. 2000 Skeletal muscle mass and distribution in 468 men and women aged 18–88 yr. J. Appl. Physiol. **89**, 81–88. (10.1152/jappl.2000.89.1.81) 10904038

[18] Abe T, Fukashiro S, Harada Y, Kawamoto K. 2001 Relationship between sprint performance and muscle fascicle length in female sprinters. J. Physiol. Anthropol. Appl. Human Sci. **20**, 141–147. (10.2114/jpa.20.141) 11385937

[19] Wright SE, Weyand PG. 2001 The application of ground force explains the energetic cost of running backward and forward. J. Exp. Biol. **204**, 1805–1815. (10.1242/jeb.204.10.1805) 11316501

[20] N.A.. 2023 World Athletics Toplists.100. See https://www.worldathletics.org/records/all-time-toplists (accessed 15 April 2023).

[21] Bruggeman GP, Koszewski D, Muller H. 1999 Biomechanical research project: Athens 1997: final report. Oxford, UK: Meyer & MeyerSport. See https://www.worldathletics.org/about-iaaf/documents/research-centre.

[22] Hay JG. 1992 The biomechanics of the triple jump: a review. J. Sports Sci. **10**, 343–378. (10.1080/02640419208729933) 1518095

[23] Nixdorf E, Mendoza L, Isele R. 2010 Scientific research project biomechanical analysis at the Berlin 2009. See https://site-canary.prod.aws.worldathletics.org/about-iaaf/documents/research-centre.

[24] Bissas A, Walker J, Tucker C, Paradisis G, Merlino S. 2017 Biomechanical report for the IAAF world championships 2017: triple jump men’s & women’s, long jump men’s & women’s, high jump men’s & women’s. IAAF World Championships Biomechanics Research Project. See https://www.worldathletics.org/about-iaaf/documents/research-centre.

[25] Bissas A, Tucker C, Merlino S. 2018 Biomechanical report for the IAAF world championships 2018: triple jump men’s & women’s, long jump men’s & women’s, high jump men’s. IAAF World Championships Biomechanics Research Project. See https://www.worldathletics.org/about-iaaf/documents/research-centre.

[26] Croskey MI, Dawson PM, Luessen AC, Marohn IE, Wright HE. 1922 The height of the center of gravity in man. Am. J. Physiol. Leg. Content **61**, 171–185. (10.1152/ajplegacy.1922.61.1.171)

[27] Olympedia. 2006 Place unknown: OlyMADMen. See https://www.olympedia.org/ (accessed 21 January 2022).

[28] 1997 Tilastopaja Oy Track and field statistics. See https://www.tilastopaja.eu/ (accessed 21 January 2022).

[29] McClelland EL, Weyand PG. 2022 Sex differences in human running performance: smaller gaps at shorter distances? J. Appl. Physiol. **133**, 876–885. (10.1152/japplphysiol.00359.2022) 35981732

[30] McClelland EL, Weyand PG. 2025 Supplementary material from: Sex performance difference in vertical and horizontal jumping. Figshare. (10.6084/m9.figshare.c.8007265) PMC12457033 40994581

